# Effect of Late-Onset Stroke Rehabilitation on Medical Morbidities and Functional Recovery: A Single-Center Observational Study

**DOI:** 10.7759/cureus.33002

**Published:** 2022-12-27

**Authors:** Pabitra Kumar Sahoo, Nehal Nehal

**Affiliations:** 1 Physical Medicine and Rehabilitation, Swami Vivekanand National Institute of Rehabilitation Training and Research (SVNIRTAR), Bairoi, IND; 2 Physical Medicine and Rehabilitation, Healthway Hospital Old Goa, Bainguinim, IND

**Keywords:** barthel index scale, cerebro-vascular accident, stroke, morbidities, neuro rehabilitation, physical medicine and rehabilitation (pm&r), stroke intervention

## Abstract

Background

The concept of focused rehabilitative care and a dedicated rehabilitation setup is fairly less known among the people of developing countries. The main objective of the study is to assess the effect of late-onset stroke rehabilitation on its overall prognosis and to see whether and how the late initiation of rehabilitation would pose a significant effect on functional recovery and adverse medical outcomes

Methods

A single-center, prospective observational study was conducted in a tertiary rehabilitation center for a duration of one and a half years. The subjects admitted to the Department of Physical Medicine and Rehabilitation were divided into three groups with respect to the onset to admission interval (OAI). Patients who got admitted within 30 days (OAI≤ 30 days) were considered the early rehabilitation group. OAI between 31 and 150 days was the late rehabilitation group and OAI of 151 days or more was the very late rehabilitation group. Barthel Index (BI), Modified Rankin Score (MRS), and 6-minute walk test (6MWT) were used as functional outcome measures at admission and discharge from indoors.

Results

Maximum complications were observed in the late rehabilitation group, i.e. 2.75±2.74, with an overall mean of 2.02±2.04 (p=0.003). Functional recovery assessed using BI shows a significant difference between the early (35.79±8.86) and late (40.62±10.5) rehabilitation groups (p= 0.0005). 6MWT at discharge showed significant improvement in the early rehabilitation group (p=0.005).

Conclusion

Early onset of rehabilitation showed better functional recovery and fewer adverse medical outcomes in stroke patients. A longer length of hospital stay (LOHS), with a mean duration of 88 ±2 days was needed for patients with higher OAIs.

## Introduction

Stroke is an acute event of cerebrovascular origin causing focal or global neurological dysfunctions lasting more than 24 hours. It is the leading cause of ensuing complications and long-term disability [1]. The Global Burden of Diseases (GBD) study of 1990 reported stroke to be one of the two frontline mortality causes [2]. With upward-trending death proportions, stroke is the second leading cause of death worldwide. The majority of stroke survivors are left with some form of disability that requires rehabilitation [2,3]. Stroke is known to have adverse outcomes, as it limits the patients’ ambulatory capacity and ultimately affects their quality of life [4]. Medical complications are an important problem after an acute stroke event and present a potential hindrance to timely recovery [3]. Generally, long-standing neurological impairments, physical inadequacies, and chronic disabilities are the residual consequences of stroke [5]. Also, psychological and emotional disturbances are often not diagnosed and are unaddressed in rehabilitation [6].

The American Stroke Association has recommended that rehabilitation can be started within two days after the onset of stroke in medically stable patients [7]. Even though animal researchers concluded that early and intensive therapy had negative effects, accentuating brain injury by overuse of the involved limb [8], the prospects of any rehabilitation program are widely time-dependent. Hence, the dictum that states "time is brain" is still accurate.

Unfortunately, prevailing studies on stroke rehabilitation outcomes are not consistent, and the effectiveness of stroke rehabilitation programs is still debatable [9]. Many studies have shown the benefits of early rehabilitation to later intervention in stroke patients [10]. But the interpretation of these studies is widely variable. For instance, the early stage of rehabilitation can begin anytime from the third to the thirtieth day. Also, this dilemma is further burdened by stroke having different types, varying severity, incomplete documentation, and variation in rehabilitation procedures for each type [9].

In contrast to developed countries, the concept of focused rehabilitative care and a dedicated rehabilitation setup is fairly less known among the Indian population. More often, after discharge from an acute care facility, unfocused therapy for a short duration of time is all that the patients get. Access to early rehabilitation following a stroke is not easily available in developing countries and a lack of awareness about the impact of early rehabilitation adds to comorbidities. However, multicenter studies with large sample sizes are required, which will help in policy-making for the rehabilitation of stroke patients. Hence, this pioneer Indian research was conducted with the main objective of studying the effect of late-onset stroke rehabilitation on its overall prognosis and seeing whether and how the late initiation of rehabilitation would pose a significant effect on the outcomes and medical complications.

## Materials and methods

This pioneering study was a prospective, observational study in a single-center, rural-based national institute of India within a period of one and a half years. A study sample size of 30 stroke patients (n=30) was calculated using the Cochrane method. Out of 41 patients admitted to the department of Physical Medicine and Rehabilitation, consecutive 30 patients meeting the inclusion criteria were recruited for the study. Scientific and ethical committee approval was taken as per national legislation and the declaration of Helsinki. The study has been approved by the institution-recognized ethical committee of JPM Rotary Eye Hospital and Research Institute vide IRB No. IECJREH/A14/2020 dated 27/02/2020. Consent was taken from all the patients before recruitment for the study. Demographic and clinical data were collected in the data collection forms. The tables were then deduced from the collected data for further consideration.

The research included cases with age > 18 yrs, first-ever stroke, either ischemic or hemorrhagic origin, hemodynamically stable, no or minimal cognitive impairment, and not having received rehabilitation services before. Subjects with ages less than 18 years, previous cerebrovascular accidents, a bilateral (B/L) cerebral lesion, cognitively impaired, incomplete/complete rehabilitation services received previously were excluded from the study. Patients with medical co-morbidities, namely, type 2 diabetes mellitus (T2DM), hypertension (HTN), hypercholesterolemia, complications of stroke such as spasticity, urinary tract infections (UTIs), pressure ulcers, falls, seizures, and shoulder pain were considered in the study. Standardized stroke rehabilitation protocols were followed in therapy departments for all patients as per institutional guidelines. However, the customization of the procedure for different groups was not supervised by the principal investigator. The optimization of hand function for the basic activities of daily living (ADL) of stroke patients was assessed and managed by the occupational therapy department. The primary criteria for discharge from the hospital were after the patient achieved the set rehabilitation goal based on the type and severity of the stroke. The outcome measures were recorded at admission and during discharge from the hospital.

Outcome measures

Rehabilitation outcomes were gauged using scales: admission and discharge Barthel Index (BI) and Modified Rankin Score (MRS) for ADL) [11-13]; 6-minute walk test (6MWT) for respiratory and ambulation outcomes [14]; Hospital Anxiety and Depression Scale (HADS) for anxiety and depression [15,16]; modified Ashworth Scale (MAS) for spasticity [17]. The National Pressure Ulcer Advisory Panel's Updated Pressure Ulcer Staging System was used to stage pressure ulcers [18].

Rehabilitation units were used to consider the intensity of stroke rehabilitation received by a patient [19]. One rehabilitation unit is 20 minutes of structured, supervised rehabilitation. Average intensity per day was thus calculated as the total units of rehabilitation during hospital stay divided by the length (days) of hospital stay (LOHS).

According to a Japanese study by Yagi and associates, an average intensity per day of 3.0 or more was considered intensive rehabilitation [19]. The majority of the population in their study received rehabilitation, with intensities ranging from 1.6-3.0 rehabilitation units per day with no compromises in functional outcomes. In view of this, the subjects in our study received similar intensity rehabilitation, as above, for most of their LOHS.

For the ease, and systematicity of the study, the subjects were divided into three groups with respect to the onset to admission interval (OAI) (time since occurrence/onset of stroke till admission to the rehabilitation unit). Patients who got admitted within or equal to 30 days (OAI≤ 30 days) were considered the early rehabilitation group. OAI between 31 and 150 days was the late rehabilitation group. OAI of 151 days and more were placed into the very late rehabilitation group.

Data analysis

The data were entered in Microsoft Excel (Microsoft Corporation, Redmond, WA). Descriptive statistics were used to measure numbers and frequencies. A multivariable logistic regression analysis using the regression function in Microsoft Excel was used. Inter-group analyses were done using analysis of variance (ANOVA). A p-value of < 0.05 was considered statistically significant.

## Results

The mean age of the study population was 58.03±13.14 years. The very late group showed the highest mean age of 68.3±9.07 years. There is a significant difference in the mean age of the three groups. The LOHS was lowest for the early rehabilitation group, which was 46.79±8.91. The very late group needed the highest LOHS of around 88±2 days.

**Figure 1:**
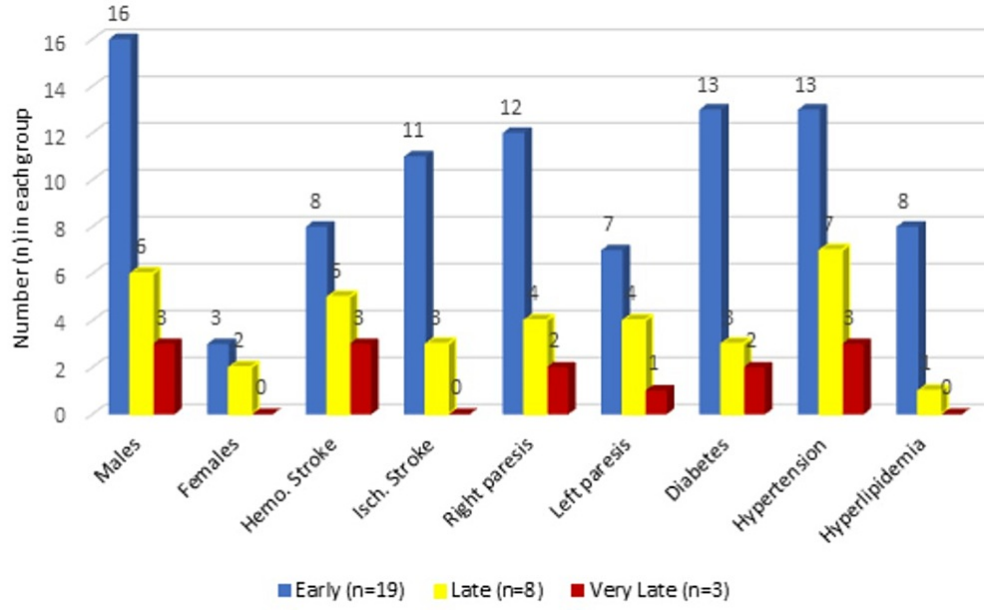
Graphical presentation of demography and co-morbidities in the study participants

Our study population had a majority of male patients. Out of the 30 patients in the study, 25 of them were male subjects and 23 of the 30 had hypertension, which was the most common co-morbidity associated with the study. Diabetes mellitus was the second most common co-morbidity associated with 18 patients. There was no significant difference in the type of stroke. There were 16 hemorrhagic stroke patients compared to 14 ischemic stroke patients. The right-sided hemiparetic presentation was more common than the left-sided presentation (n=18 vs n=12) (Table 1, Figure 1).

**Table 1:** Distribution of demographics and co-morbidities in all the three groups OAI: Onset to admission interval; OHS: The length of hospital stay

	Early (n=19)	Late (n=8)	Very late (n=3)	All patients (n=30)
Age (mean years ± SD)	56.3±13.54	58.25±12.9	68.3±9.07	58.03±13.14
OAI (mean days ± SD)	16.47±8.93	71.63±41.85	425.67±285.56	72.1±145.1
LOHS (mean days ± SD)	46.79±8.91	66.37±5.21	88±2	56.13±15.73
Sex (n)				
Male	16	06	03	25
Female	03	02	00	05
Co-morbidities (n)				
Diabetes(DM)	13	03	02	18
Hypertension (HTN)	13	07	03	23
Hyperlipidemia	08	01	00	09
Type of Stroke (n)				
Hemorrhagic	08	05	03	16
Ischemic	11	03	00	14
Side of Paresis (n)				
Right	12	04	02	18
Left	07	04	01	12

**Figure 2:**
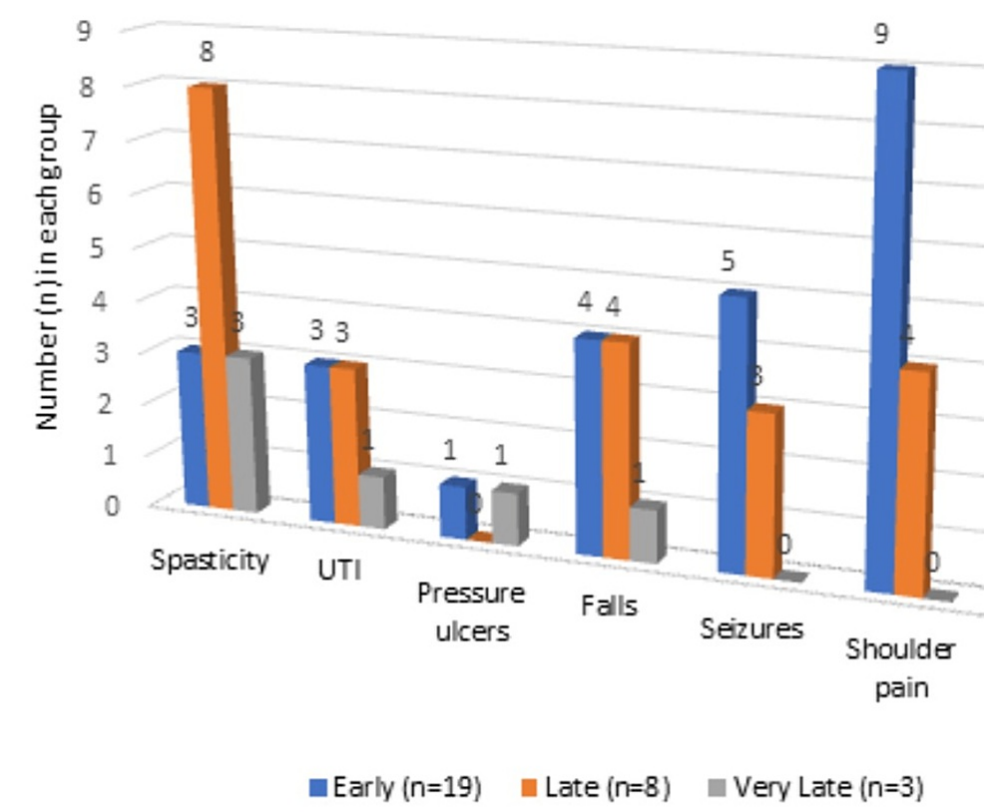
Distribution of medical complications in participants of all the 3 groups UTI: Urinary tract infection

Our study showed the maximum number of complications in the Late rehabilitation group, which was 2.75±2.74, with an overall mean of 2.02±2.04. Spasticity was the most common complication associated (n=14) overall followed by hemiplegic shoulder pain (n=13). Hemiplegic shoulder pain was also the most common complication incriminated in the early rehabilitation group (n=9) and spasticity in the late rehabilitation group (n=8). Five of the 19 subjects in the early group had at least one episode of seizure. UTI as well as falls were more prevalent in both the early and late groups. In the very late group, all three subjects had spasticity and one out of the three had a Grade III pressure injury (Table 2, Figure 2).

**Table 2:** Distribution of medical complications among all the groups

	Early (n=19)	Late (n=8)	Very late (n=3)	All patients (n=30)
Number of medical complications (mean±SD)	1.31±2.4	2.75±2.74	2±1	2.02±2.04
Spasticity	3	8	3	14
Urinary tract infections (UTIs)	3	3	1	7
Pressure ulcers	1	0	1	2
Falls	4	4	1	9
Seizures	5	3	0	8
Shoulder pain	9	4	0	13

**Figure 3:**
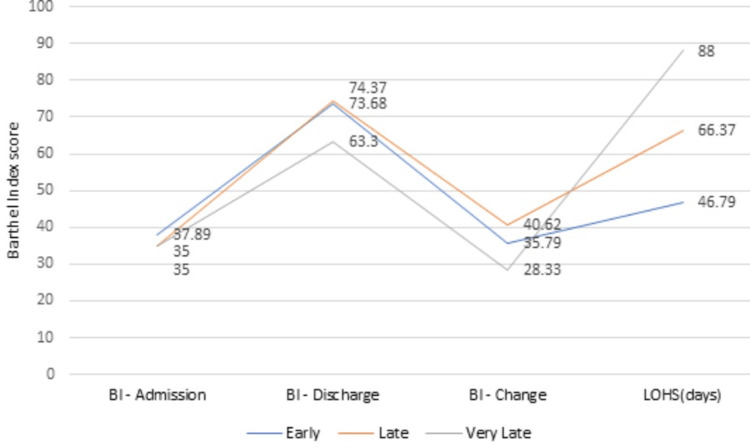
Changes in Barthel Index and comparison with length of hospital stay LOHS: Length of hospital stay

**Figure 4:**
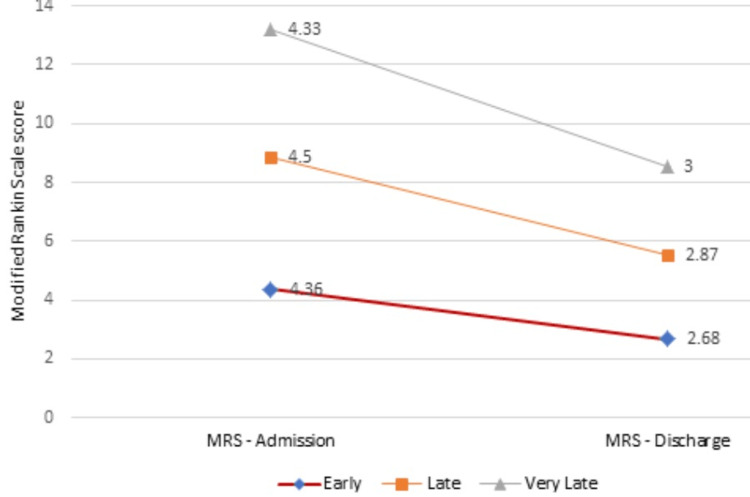
Modified Rankin Score (MRS) comparison at admission and discharge in all the groups

A mean BI at the admission of 36.83±10.12 was found in the study. The early group gave a mean admission BI score of 37.895±11.4. The mean discharge BI for the early, late, and very late groups was 73.68±8.95, 74.37±6.78, and 63.3±5.77, respectively (p=0.003). Also, the change in BI for the early rehabilitation group was 35.79±8.86, and that for the late group was 40.62±10.5 (p= 0.0005) (Tables 1, 3, Figure 3). The mean MRS on admission was 4.4±0.67 and that on discharge was 2.76±0.56 (p=0.003) (Table 4, Figure 4).

**Table 3:** Barthel Index (BI) changes in all the groups

	Early (n=19)	Late (n=8)	Very late (n=3)	All patients (n=30)	p-value
Admission BI (mean±SD)	37.895±11.4	35±7.56	35±8.6	36.83±10.12	
Discharge BI (mean±SD)	73.68±8.95	74.37±6.78	63.3±5.77	72.83±8.58	0.003
Change in BI (mean±SD)	35.79±8.86	40.62±10.5	28.33±7.63	36.33±9.55	0.0005

**Table 4:** Modified Rankin Score (MRS) comparison at admission and discharge

	Early (n=19)	Late (n=8)	Very late (n=3)	All patients (n=30)	p-value
Admission MRS (mean±SD)	4.36±0.76	4.5±0.53	4.33±0.57	4.4±0.67	0.003
Discharge MRS (mean±SD)	2.68±.67	2.87±0.35	3±0	2.76±0.56	

**Figure 5:**
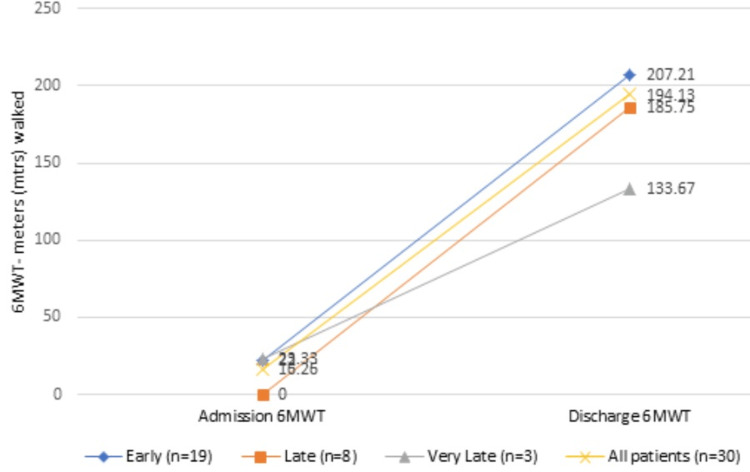
6-minute walk test (6MWT) at admission and discharge in all the groups

The mean 6MWT distance walked at admission was 16.26±43.36 meters. The subjects in the early rehabilitation group showed the highest distance walked in the discharge 6MWT results, which were 207.21±92.2 meters. The least mean distance walked was seen in the very late group, which was 133.67±29.02 meters (p=0.005) (Table 5, Figure 5).

**Table 5:** 6-minute walk test (MWT) at admission and discharge in all the groups

	Early (n=19)	Late (n=8)	Very late (n=3)	All patients (n=30)	p-value
6MWT admission (mtrs) (mean±SD)	22±52.48	0	23.33±32.14	16.26±43.36	0.005
6MWT discharge (mtrs) (mean±SD)	207.21±92.2	185.75±42.36	133.67±29.02	194.13±79.29	

**Figure 6:**
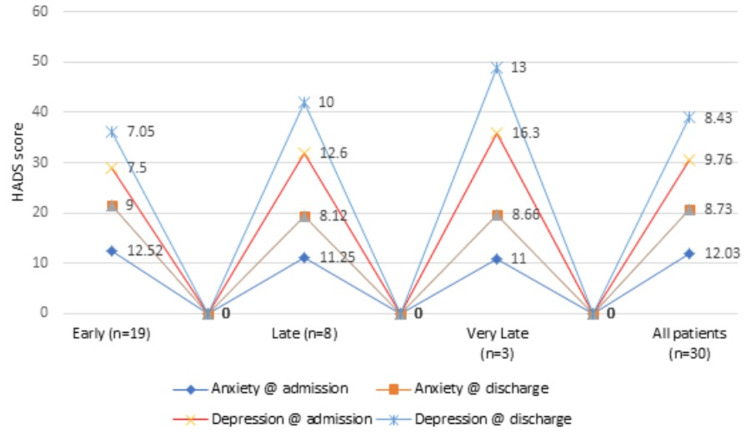
Hospital Anxiety and Depression Scale (HADS) score at admission and discharge among the groups

The early rehabilitation group showed the highest score for anxiety at admission with a mean of 12.52±2.75 compared to an overall mean score of 12.03±2.77 for anxiety. The mean score for anxiety at discharge overall was 8.73±1.6 (p=0.02). The depression score of the HADS was maximum in the very late group at admission (16.3±1.5) (Table 6, Figure 6).

**Table 6:** Hospital Anxiety and Depression Scale (HADS) score at admission and discharge among the groups

		Early (n=19)	Late (n=8)	Very late (n=3)	All patients (n=30)	p-value
HADS score Anxiety (mean±SD)	Admission	12.52±2.75	11.25±2.43	11±4	12.03±2.77	0.02
	Discharge	9±1.85	8.12±0.8	8.66±1.5	8.73±1.6	
HADS score Depression (mean±SD)	Admission	7.5±2.14	12.6±2.6	16.3±1.5	9.76±3.77	
	Discharge	7.05±1.4	10±2.56	13±1.73	8.43±2.6	

**Figure 7:**
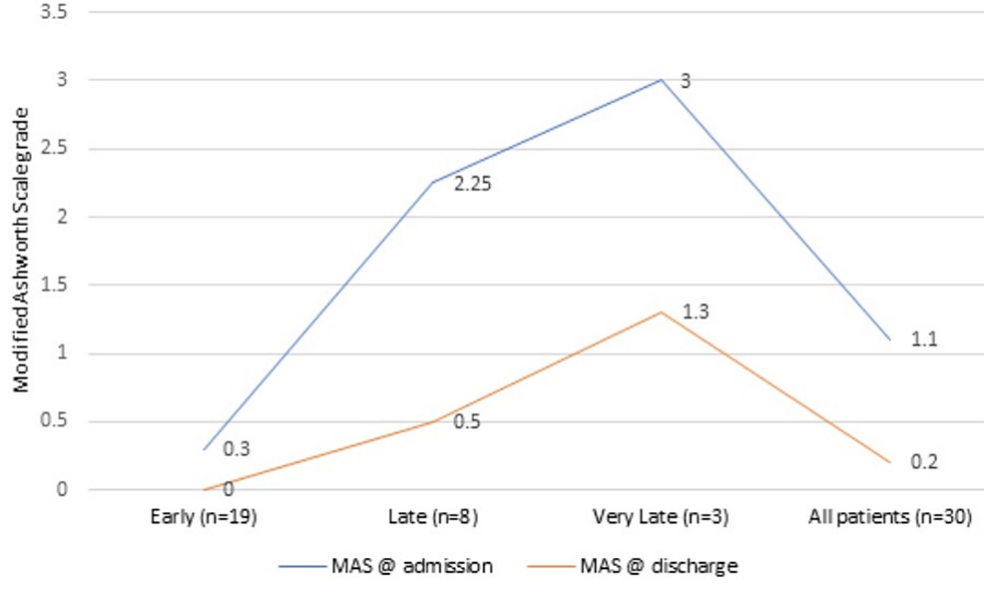
Modified Ashworth Scale (MAS) grades at admission and discharge in the three groups

All subjects in the very late rehabilitation group had Grade III MAS spasticity with a grade of 3±0, followed by the late group with a mean grade of 2.25±0.7. At discharge, all subjects in the early group were relieved from their spasticity with a Grade 0, whereas the very late group subjects had persisting spasticity of a mean grade of 1.3±0.57 (Table 7, Figure 7). The National Pressure Injury Advisory Panel (NPIAP) staging system was used for the grading of the ulcers. One patient of the early group who had an OAI of 30 days, without previous ICU stay, came with a Grade 2 ulcer. And one patient from the very late group showed a Grade 3 ulcer.

**Table 7:** Modified Ashworth Scale (MAS) grades at admission and discharge in the three groups

	Early (n=19)	Late (n=8)	Very late (n=3)	All patients (n=30)	p-value
MAS grades admission (mean±SD)	0.3±0.74	2.25±0.7	3±0	1.1±1.2	p=0.4
MAS grades discharge (mean±SD)	0	0.5±0.53	1.3±0.57	0.2±0.5	

## Discussion

The worldwide incidence of stroke varies drastically. A decrease in the incidence rate of stroke in developed western countries is seen but not in developing countries [7]. The GBD study of 1990 reported stroke to be one of the two frontline mortality causes. The past two decades have seen 26% higher stroke-related mortalities. In contrast to the developed countries, the concept of focused rehabilitative care and a dedicated rehabilitation setup is fairly less known amongst the Indian population which led us to conduct this study.

The overall mean age of our study population was around 58.03±13.14 years. The early, late, and very late rehabilitation groups had a mean age of 56.3±13.54, 58.25±12.9, and 68.3±9.07 years, respectively. Yagi et al., in their study, acquired a mean age of around 73 years [19]. Manimmanakorn and his co-workers got a mean age group of around 54 years in their stroke study [20]. A stroke rehabilitation study from London had a mean age of around 68.8 years in the <30 days group compared to 70 years in the 31-150 days group [8]. This shows us the wide range of differences in the age group of the worldwide population afflicted by stroke who need rehabilitation.

Kuptniratsaikul and his co-workers found that the median OAI was 24 days (minimum = 1, maximum = 1456 days) and 16 of these subjects were admitted after one year [6]. OAI from another study from Thailand is 53 days and 271.5 days in Brazil [21,22]. An OAI of 20 days was found in Italy and United States [23,24]. Comparatively, the mean overall mean OAI in the current study was 72.1±145.1 days. The early group showed a mean OAI of 16.47±8.93 days, the late group with 71.63±41.85 days, and the very late group showed a mean OAI of 425.67±285.56 days. Developed countries have a very low OAI, whereas developing countries have variable OAI, some of which are beyond a year. This shows the discrepancy among these countries in their stroke treatment awareness, response as well as knowledge about availing PMR facilities. 

The study we conducted showed an overall length of hospital stay (LOHS) of 56.13±15.73 days. The mean LOHS in the early rehabilitation group was 46.79±8.91 days, that for the late group was 66.37±5.21, and the very late group required a mean LOHS of 88±2 days to achieve basic ADL gains. Optimization of ADL was assessed by the occupational therapy department of the institute. The study done by Savas and his associates was the only study that considered LOHS as a variable. They divided their study population into only two groups. The mean LOHS in the <30 days OAI group was found to be 34 days and in the >30 days group, it was 30.08 days [23]. An Italian study mentioned their mean LOHS to be around 68 days overall [25].

In our study, 76.66% of the study population had hypertension, which was the major co-morbidity associated. Diabetes mellitus was the second most common co-morbidity associated with the study group followed by hyperlipidemia. Savas et al. also showed a similar percentage of co-morbidities with hypertension being the most common followed by diabetes [23]. Salter et al., Paolucci et al., and Manimmanakorn and his associates also found that hypertension was the most common co-morbidity followed by diabetes [8,20,25].

Hemorrhagic stroke was the most common type of stroke (53.33%) followed by ischemic stroke (46.66%) in our study, even though the majority of the cases in the early rehabilitation group were ischemic (57.89%). M. Siddique and co-workers, Salter et al., Susan Raju et al., and Y. Ng et al., also concluded that the ischemic strokes were more than the hemorrhagic strokes in their study group [8,15,26,27]. Safer and his associates concluded that in early-onset rehabilitation (<4 weeks), ischemic stroke was more common compared to late-onset rehabilitation(>4 weeks) where hemorrhagic stroke patients were predominant with high statistical significance [7]. This finding was very much consistent with our study.

Right-sided impairment was most commonly seen in most of the studies [8,20,23]. The study we conducted showed a similar result (60% right v/s 40% left). On the contrary, a study from Singapore showed slightly more left-sided impairment than right [26].

Considering all the patients in our study, the mean number of medical complications was 2.02±2.04. Our study showed that 23.33% of the study population had UTIs, 30% of them had fallen, and 43.33% had shoulder pain as a complication. While in a study by Janus Laszuk and co-workers, UTI was the most common complication (23.2%), followed by falls (17.9%) and shoulder pain (14.9%) [28]. Depression was seen among 43.33% of our study subjects with 75% of the late group and 100% of the very late group affected by it. This was consistent with a Turkish study that had depression as the second most common complication after shoulder pain [23].

A mean change in Barthel Index score of 29 from admission to discharge was shown in their study by Yagi and his co-workers [19]. A mean change in BI of 28.95 was shown by Kuptniratsaikul et al. while Paolucci and his associates found a change of around 46 in their research group [6,25]. Whereas, in our study, we found a mean change in BI of 36.33 with high statistical significance. Our study also showed that the late group had a slightly increased change in BI than the early rehabilitation group, which could be because of the significantly longer LOHS of the late group over the early (66.37±5.21 vs 46.79±8.91). Modified Rankin Score was used in our study with BI to fill in the missing space of BI. A mean MRS score of 2.76 at discharge was seen in our study. None of the other studies seem to have used MRS as an outcome measure with respect to stroke rehabilitation.

The majority of the subjects in the late and very late rehabilitation groups were found to be depressed at admission according to the HADS depression score (mean HADS-D score of 12.6±2.6 and 16.3±1.5, respectively) while the majority of the early rehabilitation population had anxiety at admission (mean HADS-A score of 12.52±2.75) with high statistical significance. The mean HADS-A score at admission in our study was 12.03±2.77 and the mean score of HADS-D at admission was 9.76±3.77 and that on discharge was 8.73 and 8.43, respectively. In comparison, in a study from Thailand, the mean admission HADS-A score was 7.50, the mean admission HADS-D score was 8.46, and that at discharge was much less in the normal score range [20]. This suggested that our study had a tad bit higher cases, which could probably be incriminated to the longer LOHS and increased OAI, which in turn could lead to multiple psycho-social afflictions.

The six-minute walk test (6MWT) was considered in our study as an outcome measure for ambulation. It was found that the early rehabilitation group was able to walk the longest at discharge than the late and very late groups, thus suggesting that early initiation of rehabilitation would show better functional outcomes.

Spasticity was assessed by the Modified Ashworth Scale (MAS). Our study showed that the early rehabilitation group had no spasticity at discharge while the very late group had remnant spasticity at discharge. Pressure ulcers were seen in two patients of the study group who were neglected due to socio-economic and family constraints.

Limitations

As a single-center observational study with a limited study duration, the sample size is small. A larger sample size is desirable to generalize the study result on the stroke population of developing countries. The study was set up in a rural background and hence urban statistics are unaccounted for. Although standard rehabilitation protocols were followed for all patients as per institutional guidelines for stroke rehabilitation, customization of the procedure for different groups was not supervised by the principal investigator.

Recommendation

A large sample size controlled study with a standardized and monitored rehab treatment protocol considering both urban and rural populations throughout India is the need of the hour.

Further, ground-breaking research is needed that brings about consensus regarding the exact time to start rehabilitation and the exact intervals to categorize early or late rehabilitation.

A study that would discuss the measures, and interventions taken to achieve the functional outcomes in stroke patients and their comparison at admission and discharge would add further depth to the current study.

## Conclusions

The earlier the initiation of rehabilitation, the better the functional outcomes and the lesser the medical comorbidities. The result of our study supports that the early onset of rehabilitation is related to a good recovery of ambulation and functional independent status. A longer LOHS was needed for later OAIs. Indian rural and sub-urban populations are still devoid of that early, first emergency care, as well as the equally pivotal physiatrist consult. Every case should be considered different; re-diagnosis and localization of the lesions should always precede an achievable goal setting, which will lead to better functional outcomes.
